# A New Deep Learning-Based Methodology for Video Deepfake Detection Using XGBoost

**DOI:** 10.3390/s21165413

**Published:** 2021-08-10

**Authors:** Aya Ismail, Marwa Elpeltagy, Mervat S. Zaki, Kamal Eldahshan

**Affiliations:** 1 Mathematics Department, Tanta University, Tanta 31511, Egypt; aya.ismail@science.tanta.edu.eg; 2 Systems and Computers Department, Al-Azhar University, Cairo 11884, Egypt; 3 Mathematics Department, Al-Azhar University (Girls Branch), Cairo 11884, Egypt; mervatzaki.1959@azhar.edu.eg (M.S.Z.); dahshan@gmail.com (K.E.)

**Keywords:** deepfake, YOLO, face detector, convolutional neural network, XGBoost, deepfake, fake video detection

## Abstract

Currently, face-swapping deepfake techniques are widely spread, generating a significant number of highly realistic fake videos that threaten the privacy of people and countries. Due to their devastating impacts on the world, distinguishing between real and deepfake videos has become a fundamental issue. This paper presents a new deepfake detection method: you only look once–convolutional neural network–extreme gradient boosting (YOLO-CNN-XGBoost). The YOLO face detector is employed to extract the face area from video frames, while the InceptionResNetV2 CNN is utilized to extract features from these faces. These features are fed into the XGBoost that works as a recognizer on the top level of the CNN network. The proposed method achieves 90.62% of an area under the receiver operating characteristic curve (AUC), 90.73% accuracy, 93.53% specificity, 85.39% sensitivity, 85.39% recall, 87.36% precision, and 86.36% F1-measure on the CelebDF-FaceForencics++ (c23) merged dataset. The experimental study confirms the superiority of the presented method as compared to the state-of-the-art methods.

## 1. Introduction

The growing popularity of social networks such as Facebook, Twitter, and YouTube, along with the availability of high-advanced camera cell phones, has made the generation, sharing, and editing of videos and images more accessible than before. Recently, many hyper-realistic fake images and videos created by the deepfake technique and distributed on these social networks have raised public privacy concerns. Deepfake is a deep-learning-based technique that can replace face photos of a source person by a target person in a video to create a video of the target saying or doing things said or done by the source person. Deepfake technology causes harm because it can be abused to create fake videos of leaders, defame celebrities, create chaos and confusion in financial markets by generating false news, and deceive people.

Manipulating faces in photos or videos is a critical issue that poses a threat to world security. Faces play an important role in humans interactions and biometrics-based human authentication and identification services. Thus, plausible manipulations in face frames can destroy trust in security applications and digital communications [1]. As a result, analyzing and detecting faces from photos or videos constitute a central role in detecting fakes. Several research papers have been presented in this area; facial landmark detection-based methods [2,3], Viola–Jones face detector [4], dlib detector [5], BlazeFace [6], RetinaFace [7], and multi-task convolution neural network (MTCNN) [8], to name just a few.

The first deepfake video launched in 2017 when a Reddit user transposed celebrity faces into porn videos, and consequently, several deepfake video detection methods have been presented. Some of these methods detect the temporal inconsistencies across videos’ face frames using recurrence networks, while other methods detect visual artifacts inside frames using convolution networks [9].

This paper introduces a new efficient architecture, YOLO-InceptionResNetV2-XGBoost (YIX), which discovers the visual discrepancies and artifacts within video frames and then judges whether a given video is real or a deepfake. The combination of these three methods is justified as follows: The YOLO detector proves its efficiency in object detection and face recognition systems over the state-of-the-art detectors [10,11] since it has a good trade-off between performance and speed [12,13]. Additionally, it is characterized by its ability to produce fewer false positives in the background [14], thus improving the detection method performance. In Dave et al. [15], the YOLO detector is used for detecting and counting various classes of vehicles, aiming to improve smart traffic management systems. A face detection method based on YOLO is employed for detecting the faces from the WiderFace dataset [13]. The performance achieved by this method surpasses the performance of other face detectors, and it is designed for real-time detection on mobile or embedded devices. As a result, YOLO is proposed to be used as a face detector that extracts the faces from video frames. Moreover, CNN assures its success in automatically learning the key features from images and videos. Therefore, a fine-tuned InceptionResNetV2 CNN is proposed here as a feature extractor method aiming to discover the inconsistencies in spatial information of manipulated facial video frames. Furthermore, the XGBoost model produces competitive results. It is a highly flexible and scalable machine learning model which avoids overfitting. Again, Dave et al. [15] uses the XGBoost method on the top of the YOLO vehicle detector addressing the traffic congestion problem by estimating the optimized time of the green light window. A deep-learning-based feature extraction method with the XGBoost model is employed to diagnose COVID-19 and pneumonia patients on chest X-ray images [16]. This method based on XGBoost achieves high performance compared to other machine learning methods. Traditionally, a densely connected layer with Softmax activation function is used on the top of CNN [17,18,19]. The approach adopted here is to use the XGBoost to distinguish a deepfake video from a real one. This aims to combine the advantages of both CNN and XGBoost models to improve deepfake video detection since a single model may not be powerful enough to meet the required accuracy for detecting deepfakes. Furthermore, different state-of-the-art face detection methods, CNN models, and machine learning algorithms will be explored. The newly proposed hybrid method, YIX, outperforms in all scenarios on the CelebDF-FaceForencics++ (c23) dataset. In summary, this paper introduces the following contributions:

- A new model, namely InceptionResNetV2-XGBoost, is presented to learn the spatial information and then detect the authenticity of videos. This is because deepfake videos suffer from visual artifacts and discrepancies within frames. The proposed model provides more accurate output by combining the InceptionResNetV2 as a trainable extractor that automatically extracts the informative features from video frames and XGBoost as a classifier at the top of the network to detect the deepfakes. This distinctive two-phase model assures the high reliability of feature extraction and detection.
- A YOLO face detector, an improved version of YOLO v3, is used for detecting the face regions from videos, helping to enhance the performance of detecting the deepfakes in videos.
- A comparative study for different deep-learning and classification approaches applied in the context of detecting deepfakes is introduced, in terms of AUC, accuracy, specificity, sensitivity, recall, precision, and F-measure.

The rest of the paper is organized as follows: Section 2 introduces a review of deepfake video creation and detection methods and popular existing deepfake datasets. Section 3 proposes a new architecture for detecting deepfakes in video frames. Section 4 is dedicated to the experimental results and analysis. Section 5 presents the conclusion and future work.

## 2. Literature Review

Recently, deepfake techniques gained notable popularity due to the high-quality of their generated videos and the accessibility of their applications by different users. FakeApp, Faceswap, DeepFaceLab, Faceswap Generative Adversarial Network (GAN), and DeepFake tensorflow are some of the popular deepfake face applications that are based on autoencoder-decoder and GAN architectures. The autoencoder extracts hidden features of face photos and the decoder reconstructs the face photos. For switching target and source faces, two encoder-decoder pairs with shared weights for the encoders are required, where each pair is employed to train on a face photo set. Then, the feature set of the first face is associated with the decoder of the second one to rebuild the second face from the first original face [20]. GAN consists of two deep networks, discriminator and generator, which train synchronously during the learning step. The discriminator is optimized for the sake of distinguishing between genuine and created photos, while the generator is trained to fool the discriminator from distinguishing between genuine and created photos [21].

Deepfake detection is a binary classification problem that evaluates the authenticity of videos; hence, it needs a large genuine and fake video dataset to train the model. The available deepfake video datasets are DeepFake-TIMIT [22], UADFV [23], FaceForensics++ (FF++) [17], Google/Jigsaw DeepFake Detection [24], Celeb-DeepFake (Celeb-DF) [25], Deepfake Detection Challenge (DFDC) [26], DeeperForensics-1.0 [27], and WildDeepfake [28].

Several methods have been elaborated to detect video deepfakes relying on either the visual inconsistencies and artifacts within video frames or the discrepancies in the temporal correlation. In Afchar et al. [18], the Viola–Jones face detector [4] is used to detect face regions from video frames. These faces are aligned using a facial landmark detection algorithm. Then, the MesoInception-4 and Meso-4 are employed to detect the deepfake videos on a dataset collected from the Internet. In Rossler et al. [17], a tracking method for faces is employed to track and detect face regions from video frames in the FaceForensics++ dataset, and then the XceptionNet is applied. The work in Li & Lyu [19] extracts the face regions from video frames using the dlib detector. Then, four deep learning models, ResNet152, ResNet50, VGG16, and ResNet101, are applied to discover the artifacts from face frames based on the inconsistencies in resolution between the warped face region and its surrounding context. After that, this method is validated on two deepfake video datasets, which are Deepfake-TIMIT and UADFV, and is also tested on several YouTube deepfake videos. In Nguyen et al. [29], the VGG-19 network is employed to extract the features of the detected face frames. These features are used as input to three capsule networks for detecting the authenticity of online videos collected by Afchar et al. [18]. The work in Nguyen et al. [30] creates an autoencoder based on a CNN to detect the manipulated videos in the FaceForensics++ (c23) dataset and to locate the manipulated regions. The face areas are detected and used as input to the autoencoder, which consists of encoder and Y-shaped decoder and employs a semi-supervised mode for the training process. In Dang et al. [31], the InsightFace software is used to crop out the bounding box of face frames and to mark five facial landmarks. Next, an attention layer is injected into the XceptionNet and VGG16 networks to produce attention feature maps. This helps to detect the manipulated areas in video face frames and then detect the authenticity of video. This method is trained on a large dataset, DFFD, that comprises videos created by the Deep Face Lab and FaceForencies++ video dataset and is tested on the Celeb-DF and UADFV datasets. In Li et al. [32], the face photo is cropped into small patches that are fed into ResNet-18 CNN. Then, two branches are combined; the first one learns the difference between genuine and deepfake face patches, and the second captures the inconsistencies between the face area and its surrounding region. This method is trained on Faceforensics++ and DeepfakeTIMIT datasets and is evaluated on 100 videos which are collected from YouTube. The work in Charitidis et al. [33] proposes a pre-processing approach after the face detection step to remove a large number of false positive face photos. Then, they use different CNN architectures: MesoInception4, XceptionNet, and EfficientNet-B4. This method is trained on the DFDC dataset and is evaluated on the FaceForensics++ and Celeb-DF datasets. The authors in Li et al. [25] employ several CNN architectures: InceptionV3, MesoInception4, GoogLeNet, ResNet-50, Meso4, XceptionNet, VGG19-based CapsuleNet, FWA-based Dual Spatial Pyramid (DPS), and multi-layer feed-forward network-based designed CNN. Then, these various architectures are trained on different datasets and tested on the Celeb-DF dataset. The work in Kumar et al. [34] extracts the face regions from video frames of the Celeb-DF dataset using the MTCNN and then applies the XceptionNet architecture. The authors in Khalil et al. [35] use YOLO v3 for face detection. Then, two methods of feature extraction are fused to extract the spatial information, namely local binary patterns that are based on texture and modified high-resolution network that based on CNN. After that, these spatial features are passed into capsule networks for detecting the deepfakes. This method is trained on the DFDC-Preview dataset and tested on both DFDC-Preview and Celeb-DF datasets. In Wodajo et al. [36], the face regions are extracted using three deep learning face detection methods: BlazeFace, MTCNN, and face_recognition. Then, a stack of convolution blocks is used as a feature extractor followed by a vision-transformer that is based on an attention mechanism for detecting the authenticity of the DFDC videos dataset.

The work in Güera & Delp [20] suggests using the temporal architecture, InceptionV3-LSTM, for detecting the authenticity of 600 videos. The InceptionV3 network is employed to extract the frame features, and then the features of consecutive frames are associated and fed as input into the LSTM. In Li et al. [37], the face frames are extracted using the dlib detector and the facial landmarks are marked. Then, the face frames are aligned using alignment algorithms, and eye regions are cropped into a sequence of eye frames and passed into the temporal pipeline VGG16-LSTM. The VGG16-LSTM is used to learn the temporal patterns of eye blinking. After that, this method is evaluated on 49 online videos and their corresponding generated deepfake videos. Sabir et al. [38] employ face masks [17] for detecting the face region from frames. Then, these face frames are aligned and passed into two temporal models: the ResNet-GRU (gated recurrent unit) model and DenseNet-GRU. These models are used to learn the spatial-temporal features and then detect the authenticity of the FF++ videos dataset. In Wubet [39], the VGG16 and ResNet-50 models are used to extract the eye frames’ features and to classify the states of the eye: closed or opened. The LSTM temporal model is then employed for detecting fake videos of the UADFV dataset depending on the eye blinking speed. Singh et al. [40] extract the face regions from video frames using the MobileNet-SSD detector. Then, four deep learning architectures are used with a time-distributed layer and LSTM model, namely EfficientNet-B1, InceptionV3, XceptionNet, and EfficientNet-B3. This learns the spatial-temporal features and detects the authenticity of the DFDC dataset videos. Jiang et al. [27] employ the ResNet50-LSTM and Inflated 3D ConvNet models to learn the spatial-temporal features and then detect the authenticity of the DeeperForensics-1.0 videos dataset. In De Lima et al. [41], the face regions are detected using the RetinaFace, and then the 3D CNNs are applied to learn the spatial-temporal features and detect the fake in the Celeb-DF videos dataset. In Masi et al. [42], the face frames are aligned, and then two DenseBlocks models are applied to merge the information from the frequency and color domains. These blocks are followed by Bi-LSTM to learn the temporal information and detect the deepfake videos. This method is trained on the FF++ dataset and tested on the Celeb-DF dataset. Montserrat et al. [43] use the MTCNN face detector to extract the face regions from frames. Then, the EfficientNet-B5 is applied to extract the prominent features, followed by the automatic face weighting layer and GRU to predict the authenticity of the DFDC videos dataset. Mehra [44] uses the Mobilenet SSD face detector to detect the face region from video frames that are selected using the frame selection method. Then, a part of the VGG19 network is applied followed by Capsule networks and LSTM to detect the deepfakes in the DFDC video dataset. The Capsule networks are used as a feature extractor to learn the spatial discrepancies within frames, and LSTM is employed to take these feature sequences and identify the temporal discrepancies across frames. The work in Nguyen et al. [45] extracts the face areas from the video frames of FF++ and VidTIMIT datasets. Then, the 3D convolutional neural network is applied to capture the spatial-temporal features and detect deepfake videos.

## 3. The Proposed Methodology

**Figure 1:**
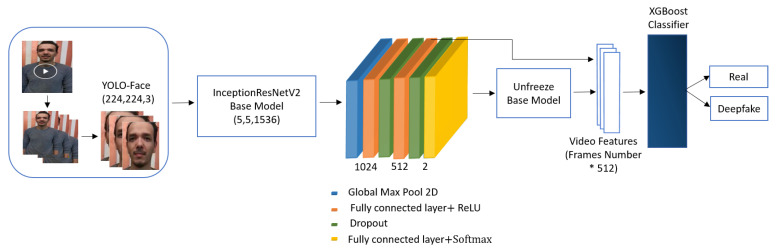
Deepfake videos detection system architecture of the proposed YIX model.

The proposed scheme introduces an effective method for detecting deepfakes in videos. Figure 1 shows the system architecture of the suggested deepfake video detection scheme. As seen in Figure 1, the suggested method employed the YOLO face detector to detect faces from video frames. The discriminant spatial-visual features are extracted using the InceptionResNetV2 CNN model. These features help to explore the visual artifacts within video frames and are then distributed into the XGBoost classifier to differentiate between genuine and deepfake videos. The proposed scheme can be explained in detail as follows.

### 3.1. Pre-Processing Stage

The frames are extracted from videos. Faces have great importance in current manipulation methods; therefore, deriving the face area features should be a major function. The YOLO face detector is employed to detect faces from video frames. Since the YOLO detector is trained to detect tight bounding boxes of faces, we increase the detected bounding box size of the face by 22% relative to its region. This produces more area around the face, helping to detect the deepfakes. Then, these face photos are scaled to the size of 224 × 224.

YOLO [46] is the first CNN-based detector that employs one neural network to predict the bounding boxes and class probabilities from the input photos in one shot. It divides the photo into grid cells of size M×M where each cell attempts to detect the object that falls in its center. Then, each grid cell predicts the coordinate values of bounding boxes, confidence scores, and classification outcomes for those boxes. YOLO v3 is a new version of YOLO that is based on the darknet-53 network which is a combination of two networks: darknet-19 and ResNet-34. This network is composed of 3 × 3 and 1 × 1 successive convolutional layers and skip connections. It is more efficient and powerful than ResNet and darknet-19. The YOLO face detector architecture [47] is based on YOLO v3. It is established by improving the darknet-53 backbone network via increasing the number of layers of the first two residual blocks to gain more sufficient small-scale face features. In addition, the anchor boxes and loss function are improved appropriately for face detection since the anchor boxes’ ratios and scales are significant hyperparameters in object detection. The anchor shapes used for face detection are (3, 3), (4, 5), (6, 8), (30, 61), (45, 62), (59, 119), (90, 116), (156, 198), and (326, 373) [47].

### 3.2. Spatial-Visual Features Extraction Stage

The discriminant spatial features for each face photo are derived using one of the pre-trained CNN models: InceptionResNetV2. The InceptionResNetV2 is an Inception-style network that uses residual connections rather than filter concatenation. The InceptionResNet block comprises multiple convolution layers of different sizes that are merged using residual connections [48]. InceptionResNetV2 network is utilized after dismissing its final dense layer as a base model, and it has been pre-trained on ImageNet weights. Then, the base model is fine-tuned with a global maximum pool layer to only pass the valid information. Afterwards, a couple of fully connected layers together with a rectified linear activation function (ReLU) are added, where each layer is followed by a dropout layer. This dropout layer is used to prevent overfitting during training [49]. Additionally, a fully connected layer is added as an output layer. Since the ImageNet dataset has 1000 distinct classes of photos, the base model is retrained with face information to make the first layers concentrate on the facial features. The description of layers for the proposed model are shown in Table 1.

### 3.3. XGBoost Based Deepfake Detection Stage

The spatial-visual features are fed to the XGBoost recognizer to distinguish between real and deepfake videos. The XGBoost is a scalable and optimized version of the gradient boosting algorithm which employs more accurate approximations to discover the optimal tree model. It is created to be flexible and highly efficient. It presents a parallel tree boosting that solves numerous data science problems in a fast and precise way [50,51].

The XGBoost utilizes an ensemble of N classification and regression trees (CARTs). The final prediction outcome is the total of all prediction scores for each of these trees. The formula of XGBoost model is given by

(1) $$\hat{y_{i}}=\overset{N}{\underset{n=1}{\sum }}f_{n}\left(x_{i}\right)$$

where $x_{i},\;i=1,\ldots ,m$, represents the members of the training dataset, $y_{i}$ represents the class labels corresponding to these members, $f_{n}\in F$ represents the leaf score for the $n^{\mathrm{th}}$ tree, and F represents the set of all CARTs. The objective function (obj) formula to be optimized is defined as follows:

(2) $$\mathrm{obj}=\overset{m}{\underset{i=1}{\sum }}l\left(\hat{y_{i}},y_{i}\right)+\overset{N}{\underset{n=1}{\sum }}Ω\left(f_{n}\right)$$

The term l represents the training differentiable loss function measuring the differences between the prediction and target values, $\hat{y_{i}}$ and $y_{i}$. The regularization term Ω is used to control the model complexity, helping to avoid overfitting. Its formula is defined as follows:

(3) $$Ω\left(f\right)=\gamma T+\frac{1}{2}\lambda \;\overset{T}{\underset{h=1}{\sum }}w_{h}^{2}$$

where γ and λ represent the constants that control the degree of regularization, T represents the number of leaves in the tree, and $w_{h}$ represents the weight score of the leaf h.

## 4. Experiments and Results

The efficacy of the proposed scheme is evaluated based on the conducted experiments. The suggested deepfake detection method is trained using the CelebDF-FaceForencies++ (c23) dataset, and the evaluation is conducted using the Celeb-DF test set. The training dataset is divided randomly into two sets: training and validation sets. The image pixel values are scaled into the range between −1 and 1.

### 4.1. Dataset

Since combining two datasets produces a more diversifiable videos dataset, matching those that may be faced in the real world, the CelebDF-FaceForencics++ (c23) dataset has been employed to evaluate the suggested model’s robustness. This aims to enhance the generalizability of the deepfake video detection model in real-world scenarios. The CelebDF-FaceForencics++ (c23) dataset is composed of two popular datasets: Celeb-DF and FaceForencics++ (c23).

The FaceForencics++ dataset is created depending on four manipulation techniques, including Deepfakes, for automatically generating fake faces in videos. It consists of 1000 genuine videos and 1000 fake videos for each manipulation technique. It has been generated in three various compression factors, which are raw, light (c23), and high (c40). The Celeb-DF dataset contains 890 real videos selected from interviews on YouTube and 5639 deepfake videos generated by an amended deepfake synthesis algorithm. It is originally separated into 5299/712 as a training set and 518 (340/178) as a testing set for deepfake and authentic videos. It is considered a more challenging and realistic dataset due to its manipulation procedure which creates few artifacts.

To train the proposed model, the 712 authentic Celeb-DF videos and 712 fake videos selected randomly from the Celeb-DF fake videos are employed. These authentic and fake Celeb-DF videos are merged with 712 real and 712 deepfake videos, which are selected randomly from the FaceForencics++ (c23) dataset. The Celeb-DF test set is especially utilized for testing due to its fake videos being created using an enhanced deepfake algorithm [25]. This algorithm produces high-quality visual videos closely matching those in the real world. Table 2 shows the distributions of training and testing sets of the CelebDF-FaceForencics++ (c23).

### 4.2. Evaluation Measures

The AUC is a popular evaluation metric utilized to assess the usefulness of the suggested deepfake video detection method. It is a single scalar value that evaluates the performance of a binary classifier [52]. The AUC is a robust measure because its calculation depends on the complete receiver operating characteristic (ROC) curve across all classification thresholds. It measures the whole two-dimensional region underneath the ROC curve from (0, 0) to (1, 1). The ROC curve represents a trade-off between true positives and false positives. It created by plotting false positive and true positive rates on X and Y axes, respectively [53]. The higher the AUC value, the better the model is at judging the video’s authenticity. In addition, six more measures are employed to evaluate the proposed model performance, which are accuracy, specificity, sensitivity, recall, precision, and F-measure. The equations of these evaluation measures are defined as follows [53].

(4) $$\mathrm{accuracy}=\frac{\;\mathrm{number}\;\mathrm{of}\;\mathrm{true}\;\mathrm{negatives}+\mathrm{number}\;\mathrm{of}\;\mathrm{true}\;\mathrm{positives}\;}{\mathrm{total}\;\mathrm{number}\;\mathrm{of}\;\mathrm{samples}\;}$$

(5) $$\mathrm{specificity}=\frac{\mathrm{number}\;\mathrm{of}\;\mathrm{true}\;\mathrm{negatives}}{\;\mathrm{number}\;\mathrm{of}\;\mathrm{true}\;\mathrm{negatives}+\mathrm{number}\;\mathrm{of}\;\mathrm{false}\;\mathrm{positives}\;}$$

(6) $$\mathrm{sensitivity}=\frac{\mathrm{number}\;\mathrm{of}\;\mathrm{true}\;\mathrm{positives}\;}{\;\mathrm{number}\;\mathrm{of}\;\mathrm{false}\;\mathrm{negatives}+\mathrm{number}\;\mathrm{of}\;\mathrm{true}\;\mathrm{positives}\;}$$

(7) $$\mathrm{recall}=\frac{\mathrm{number}\;\mathrm{of}\;\mathrm{true}\;\mathrm{positives}\;}{\;\mathrm{number}\;\mathrm{of}\;\mathrm{false}\;\mathrm{negatives}+\mathrm{number}\;\mathrm{of}\;\mathrm{true}\;\mathrm{positives}\;}$$

(8) $$\mathrm{precision}=\frac{\mathrm{number}\;\mathrm{of}\;\mathrm{true}\;\mathrm{positives}\;}{\;\mathrm{number}\;\mathrm{of}\;\mathrm{false}\;\mathrm{positives}+\mathrm{number}\;\mathrm{of}\;\mathrm{true}\;\mathrm{positives}\;}$$

(9) $$F-\mathrm{measure}=2\times \frac{\mathrm{recall}\times \mathrm{precision}\;}{\mathrm{recall}+\mathrm{precision}\;}$$

### 4.3. Experimental Results and Discussion

To justify the selection of the suggested model blocks and ensure its effectiveness, the experiments have been performed as follows.

Experiment 1: In this experiment, the selection of the convolutional neural network model is justified by comparing it with the state-of-the-art models. Different architectures of convolutional neural networks, especially InceptionResNetV2, ResNet152V2 [54], ResNet152 [54], XceptionNet [55], and EfficientNet-B5 [56], are employed as base models that were pre-trained using ImageNet weights. These architectures are fine-tuned before being applied to the CelebDF-FaceForencies++ (c23) dataset. The global max pool two-dimensional layer is added, and it is followed by two fully connected layers with 1024 and 512 units, respectively. The fully connected layers are associated with a ReLU activation function, and each of these layers is followed by a dropout layer with 0.5 and 0.2 probability rates, respectively. Next, a fully connected layer with two units together with a Softmax activation function is added. These base models are re-trained to concentrate on learning face information. Moreover, the Nesterov-accelerated adaptive moment estimation (Nadam) optimizer [57] is employed together with a learning rate of 0.00002 and schedule decay of 0.00004 for updating the weight parameters in order to minimize the difference between the actual and predicted outputs. Furthermore, the cross-entropy loss is used as a loss function on the suggested model to measure the efficiency of the CNN model. As shown from Table 3, the InceptionResNetV2-XGBoost-based method outperforms other CNN-XGBoost-based methods using the AUC evaluation metric. Furthermore, the InceptionResNetV2-SVM (support vector machine)-based method achieves outstanding performance compared to other CNN-SVM-based methods using the AUC metric.

Experiment 2: In this experiment, five scenarios are accomplished. The first one applies the XGBoost classifier on the features of videos that are extracted from the CNN model to distinguish between genuine and deepfake videos. The parameters used in XGBoost are Learning_rate, M_estimators, Max_depth, Min_child_weight, Gamma, Subsample, Colsample_bytree, Objective, Num_class, and Nthread [51] with values 0.1, 100, 5, 1, 0, 0.8, 0.8, Softmax, 2, and 4, respectively. Additionally, the multiclass log loss (mlogloss) is the evaluation metric used to evaluate the accuracy of the XGBoost model on the validation set.

The second one applies the SVM classifier [59] to the CNN features of videos. The SVM maps the input vector of data to a higher dimensional feature space where a maximal margin hyperplane is constructed. The parameters used in SVM are the kernel and the regularization C [60] with values radial basis function (‘rbf’) and 10,000, respectively. The third one uses the fully connected (dense) layer with Softmax activation function on the top of CNN directly to differentiate between real and fake videos. The fourth scenario utilizes the random forest (RF) classifier on the key video features that are extracted from the CNN. The RF is an ensemble learning method that utilizes the average prediction score of a single tree within a combination of several decision trees [61]. The parameters employed in the RF are n_estimators and random_state with values 100 and 42, respectively. The fifth one uses the AdaBoost classifier on top of the CNN features. The AdaBoost is a boosting ensemble sequential learning method in which every weak classifier is tweaked depending on the misclassified instances from all previous classifiers [62]. Its final decision is the weighted sum of outcome scores from a combination of the final classifiers. In AdaBoost, the decision tree is used as a base classifier, and the n_estimators parameter is utilized with a value of 50.

As seen from Table 3, the proposed method recorded the highest performance. Whilst the YIX proposed hybrid method registered an AUC score of 90.62%, the YOLO-InceptionResNetV2 based SVM, DenseLayer, RF, and AdaBoost methods recorded 86.05%, 61.31%, 78.30%, and 76.04%, respectively. This means that the XGBoost-based proposed method outperforms other methods based on either the dense layer classifier with Softmax function or the traditional machine learning methods, SVM, RF, and AdaBoost. In addition, as shown in Table 3, the AUC results for the XGBoost classifier on the CelebDF-FaceForencies++ (c23) dataset exceed that for the SVM classifier on all conducted experiments. This is due to the fact that SVM has fewer advantages when the training dataset size is big enough. Moreover, XGBoost is an ensemble learning method that utilizes several decision trees to make its decision. Thus, it gains its power by looping itself M_estimators times. This amount of decision trees helps XGBoost fit the training data more flexibly and learn more information from the data. Additionally, the XGBoost method controls both the number and weight of leaves in each tree using the regularization term to avoid overfitting. This enhances the model applicability outside the training data.

Experiment 3: In this experiment, the YOLO face detector is compared with three of the popular state-of-the-art face detectors: dlib [5], BlazeFace [6], and MTCNN [8]. The dlib face detector, frontal_face_detector, uses the histograms of oriented gradient (HOG) and SVM for face detection. The BlazeFace detector accepts input photos of shape 128 × 128 and uses depthwise separable convolutions to detect the face regions. The MTCNN face detector rescales the input photo into different sizes and then uses a three-stage cascaded structure to detect the face areas. These three popular detectors accompanied with the proposed YOLO based one are applied to the CelebDF-FaceForencies++ (c23) video dataset. The YOLO face detector-based proposed method performs better than dlib, BlazeFace, and MTCNN face detectors as shown in Table 3. This is because the YOLO face detector produces fewer false-positive instances compared to the other three detectors. Whilst the YIX registered an AUC score of 90.62%, the MTCNN-InceptionResNetV2-XGBoost, dlib-InceptionResNetV2-XGBoost, and BlazeFace-InceptionResNetV2-XGBoost methods recorded 86.13%, 82.89%, and 84.95%, respectively.

**Figure 2:**
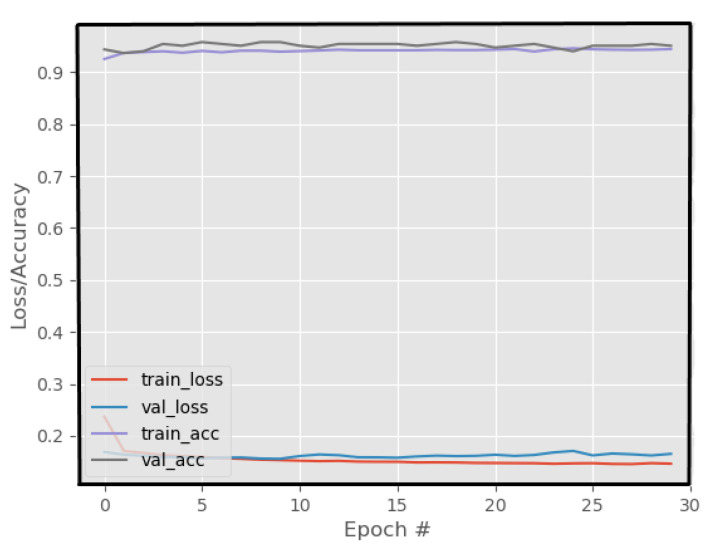
The loss and accuracy curves of the proposed model on the CelebDF-FaceForencies++ (c23) dataset.

**Figure 3:**
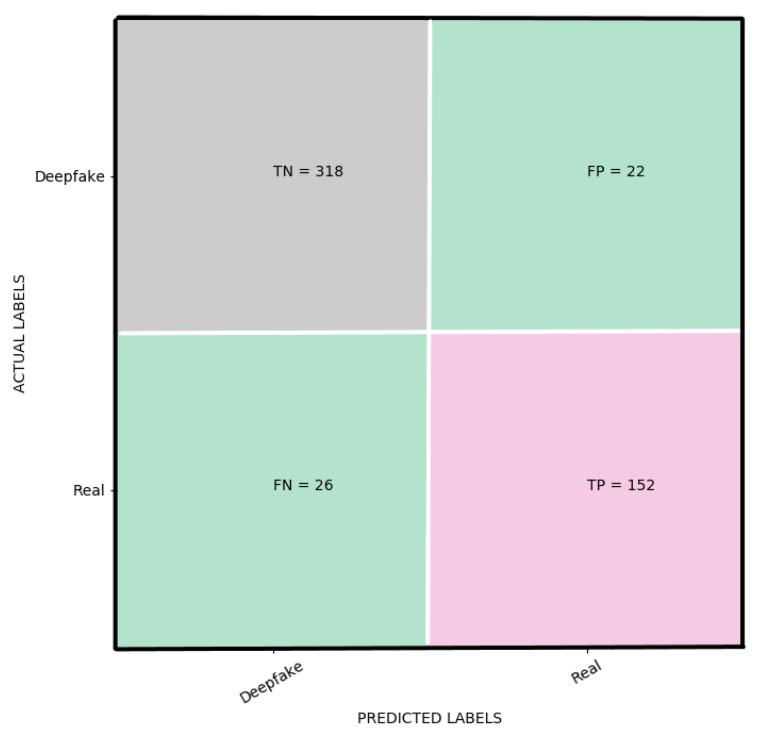
The confusion matrix for the proposed model.

**Figure 4:**
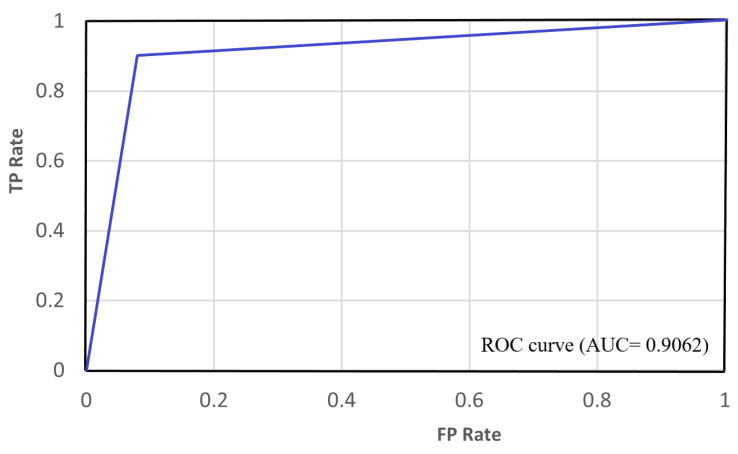
The AUC curve for the proposed model.

The loss and accuracy curves for the suggested YIX model on the training and validation sets of the CelebDF-FaceForencies++ (c23) dataset are shown in Figure 2, and Figure 3 shows the confusion matrix of the suggested model for detecting the deepfakes on the Celeb-DF test set. Additionally, Figure 4 shows the AUC curve corresponding to the performance of the suggested model. From Figure 4, it is clear that the ROC curve is close to the top-left corner which demonstrates a high performance by the proposed YIX model. As can be seen from Table 4, the proposed model yields 90.73% accuracy, 93.53% specificity, 85.39% sensitivity, 85.39% recall, 87.36% precision, and 86.36% F1-measure, respectively.

**Figure 5:**
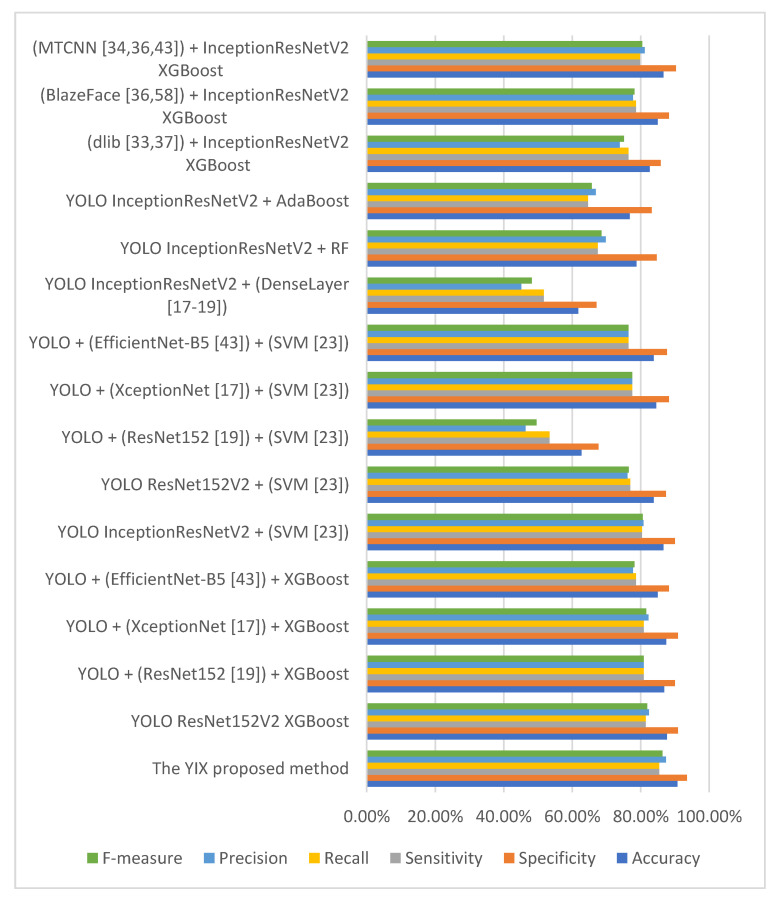
Performance of the proposed model compared to state-of-the-art models on the CelebDF FaceForencies++ (c23) merged dataset.

Moreover, the AUC-based comparative analyses of the suggested model with state-of-the-art models for training the CelebDF-FaceForencies++ (c23) dataset and testing on the Celeb-DF test set are presented in Table 3. Figure 5 shows the results of evaluation measures for comparing the proposed YIX model with state-of-the-art models on the CelebDF-FaceForencies++ (c23) dataset. The experimental results show that the suggested YIX model has yielded a higher performance level compared to the state-of-the-art models.

The experiments were conducted on Windows 10 and an HP laptop, OMEN 15-dh0xxx, that has an Intel (R) Core (TM) i7-9750H CPU-16 GB and an RTX 2060 GPU-6 GB. The Python programming language, version 3.7.4, was utilized to implement the proposed model. Keras, Tensorflow, OpenCV, Sklearn, Xgboost, Numpy, Random, OS, and PIL are some of the libraries in Python which were employed for achieving the suggested model.

## 5. Conclusions and Future Work

In this work, a new methodology for detecting deepfakes is introduced. This methodology employs the YOLO face detector to extract face areas from video frames. InceptionResNetV2 CNN is used to extract the discriminant spatial features of these faces, helping to discover the visual artifacts within the video frames. These visual features are distributed into the XGBoost classifier to distinguish between real and deepfake videos. A merged dataset is employed for the model evaluations, namely CelebDF-FaceForencics++ (c23). This dataset is based on combining two popular datasets, Celeb-DF and FaceForencies++ (c23). The suggested method achieves a high detection score based on the evaluation metrics. It records 90.62% of AUC score, 90.73% accuracy, 93.53% specificity, 85.39% sensitivity, 85.39% recall, 87.36% precision, and 86.36% F1-measure. The comparative analyses proved that the proposed method outperforms the state-of-the-art methods.

Since deepfake video creation techniques develop continuously, more efforts are needed to improve the existing detection methods. We intend to use different detectors that showed outstanding performance in object detection for face detection. Furthermore, we also intend to build a more robust deep-learning-based deepfake detection method, aiming to keep up with advances in the deepfake generation process.

## Figures and Tables

**Table 1:** Layer details of the proposed model.

**Layer (Type)**	**Output Shape**	**Parameters Number**
InceptionResNetV2 (Model)	(None, 5,5,1536)	54,336,736
global_max_pooling2d (GlobalMax)	(None, 1536)	0
dense (Dense)	(None, 1024)	1,573,888
dropout_1 (Dropout)	(None, 1024)	0
dense_1 (Dense)	(None, 512)	524,800
dropout_2 (Dropout)	(None, 512)	0
**Dense_2 (Dense)**	**(None, 2)**	1026
**Total number of parameters: 56,436,450** **Number of trainable parameters: 56,375,906** **Number of non-trainable parameters: 60,544**		

**Table 2:** Authentic and fake video numbers for distributions of training and testing sets of the CelebDF-FaceForencics++ (c23).

Training Set	Testing Set
2848 = (712 authentic-Celeb +712 deepfake-Celeb (by random selection) +712 authentic-FF++ (c23) +712 deepfake-FF++ (c23))	518 = (340 deepfake-Celeb +178 authentic-Celeb)

**Table 3:** Comparative analysis of the proposed model with state-of-the-art models on the CelebDF-FaceForencies++ (c23) videos dataset.

Method	AUC Test Result
**The** YIX **proposed method**	90.62%
**YOLO ResNet152V2 XGBoost **	87.78%
**YOLO** + (**ResNet152** [19]) + **XGBoost**	86.11%
**YOLO** + (**XceptionNet** [17]) + **XGBoost**	87.09%
**YOLO** + (**EfficientNet-B5** [43]) + **XGBoost**	84.83%
**YOLO InceptionResNetV2** + (**SVM** [23])	86.05%
**YOLO ResNet152V2** + (**SVM** [23])	83.43%
**YOLO** + (**ResNet152** [19]) + (**SVM** [23])	62.65%
**YOLO** + (**XceptionNet** [17]) + (**SVM** [23])	84.19%
**YOLO** + (**EfficientNet-B5** [43]) + (**SVM** [23])	83.64%
Y**OLO InceptionResNetV2** + (DenseLayer [17,18,19])	61.31%
YOLO InceptionResNetV2 + RF	78.30%
YOLO InceptionResNetV2 + AdaBoost	76.04%
(dlib [33,37]) + InceptionResNetV2 XGBoost	82.89%
(BlazeFace [36,58]) + InceptionResNetV2 XGBoost	84.95%
(**MTCNN** [34,36,43]) + **InceptionResNetV2 XGBoost**	86.13%

**Table 4:** The proposed model performance.

	Accuracy	Specificity	Sensitivity	Recall	Precision	F1-Measure
**YOLO InceptionResNetV2 XGBoost** (YIX)	0.9073	0.9353	0.8539	0.8539	0.8736	0.8636

## Data Availability

Datasets available online: https://github.com/ondyari/FaceForensics/tree/original and https://github.com/yuezunli/celeb-deepfakeforensics (accessed on 15 June 2021).

## References

[1] Akhtar Z., Dasgupta D., Banerjee B. Face Authenticity: An overview of face manipulation generation, detection and recognition. Proceedings of the International Conference on Communication and Information Processing (ICCIP).

[2] Vezzetti E., Marcolin F., Tornincasa S., Maroso P. (2016). Application of geometry to rgb images for facial landmark localisation-a preliminary approach. Int. J. Biom..

[3] Zhang Z., Zhang W., Liu J., Tang X. (2014). Multiview facial landmark localization in RGB-D images via hierarchical regression with binary patterns. IEEE Trans. Circuits Syst. Video Technol..

[4] Viola P., Jones M. Rapid object detection using a boosted cascade of simple features. Proceedings of the 2001 IEEE Computer Society Conference on Computer Vision and Pattern Recognition, (CVPR 2001).

[5] King D.E. (2009). Dlib-ml: A machine learning toolkit. J. Mach. Learn. Res..

[6] Bazarevsky V., Kartynnik Y., Vakunov A., Raveendran K., Grundmann M. (2019). Blazeface: Sub-millisecond neural face detection on mobile gpus. arXiv.

[7] Deng J., Guo J., Zhou Y., Yu J., Kotsia I., Zafeiriou S. (2019). Retinaface: Single-stage dense face localisation in the wild. arXiv.

[8] Zhang K., Zhang Z., Li Z., Qiao Y. (2016). Joint face detection and alignment using multitask cascaded convolutional networks. IEEE Signal Process. Lett..

[9] Nguyen T.T., Nguyen C.M., Nguyen D.T., Nguyen D.T., Nahavandi S. (2019). Deep learning for deepfakes creation and detection. arXiv.

[10] Hui K., Wang J., He H., Ip W.H. (2021). A multilevel single stage network for face detection. Wirel. Commun. Mob. Comput..

[11] Garg D., Goel P., Pandya S., Ganatra A., Kotecha K. A deep learning approach for face detection using YOLO. Proceedings of the 2018 IEEE Punecon.

[12] He Y. (2016). Object Detection with YOLO on Artwork Dataset. Adv. Comput. Vis. Jiaotong Univ..

[13] Qi D., Tan W., Yao Q., Liu J. (2021). YOLO5Face: Why reinventing a face detector. arXiv.

[14] Aralikatti A., Appalla J., Kushal S., Naveen G.S., Lokesh S., Jayasri B.S. Real-time object detection and face recognition system to assist the visually impaired. Proceedings of the First International Conference on Advances in Physical Sciences and Materials.

[15] Dave P., Chandarana A., Goel P., Ganatra A. (2021). An amalgamation of YOLOv4 and XGBoost for next-gen smart traffic management system. PeerJ Comput. Sci..

[16] Kumar R., Arora R., Bansal V., Sahayasheela V.J., Buckchash H., Imran J., Narayanan N., Pandian G.N., Raman B. (2020). Accurate prediction of COVID-19 using chest X-Ray images through deep feature learning model with SMOTE and machine learning classifiers. MedRxiv.

[17] Rossler A., Cozzolino D., Verdoliva L., Riess C., Thies J., Nießner M. Faceforensics++: Learning to detect manipulated facial images. Proceedings of the IEEE/CVF International Conference on Computer Vision.

[18] Afchar D., Nozick V., Yamagishi J., Echizen I. Mesonet: A compact facial video forgery detection network. Proceedings of the 2018 IEEE International Workshop on Information Forensics and Security (WIFS).

[19] Li Y., Lyu S. (2018). Exposing deepfake videos by detecting face warping artifacts. arXiv.

[20] Güera D., Delp E.J. Deepfake video detection using recurrent neural networks. Proceedings of the 2018 15th IEEE International Conference on Advanced Video and Signal Based Surveillance (AVSS).

[21] Wang Z., She Q., Ward T.E. (2019). Generative adversarial networks in computer vision: A survey and taxonomy. arXiv.

[22] Korshunov P., Marcel S. (2018). Deepfakes: A new threat to face recognition? assessment and detection. arXiv.

[23] Yang X., Li Y., Lyu S. Exposing deep fakes using inconsistent head poses. Proceedings of the ICASSP 2019–2019 IEEE International Conference on Acoustics, Speech and Signal Processing (ICASSP).

[24] Dufour N., Gully A. (2019). Contributing data to deepfake detection research. Google AI Blog.

[25] Li Y., Yang X., Sun P., Qi H., Lyu S. Celeb-df: A large-scale challenging dataset for deepfake forensics. Proceedings of the IEEE/CVF Conference on Computer Vision and Pattern Recognition.

[26] Dolhansky B., Howes R., Pflaum B., Baram N., Ferrer C.C. (2020). The deepfake detection challenge (dfdc) dataset. arXiv.

[27] Jiang L., Li R., Wu W., Qian C., Loy C.C. Deeperforensics-1.0: A large-scale dataset for real-world face forgery detection. Proceedings of the IEEE/CVF Conference on Computer Vision and Pattern Recognition.

[28] Zi B., Chang M., Chen J., Ma X., Jiang Y.G. WildDeepfake: A challenging real-world dataset for deepfake detection. Proceedings of the 28th ACM International Conference on Multimedia.

[29] Nguyen H.H., Yamagishi J., Echizen I. Capsule-forensics: Using capsule networks to detect forged images and videos. Proceedings of the ICASSP 2019–2019 IEEE International Conference on Acoustics, Speech and Signal Processing (ICASSP).

[30] Nguyen H.H., Fang F., Yamagishi J., Echizen I. Multi-task learning for detecting and segmenting manipulated facial images and videos. Proceedings of the 2019 IEEE 10th International Conference on Biometrics Theory, Applications and Systems (BTAS).

[31] Dang H., Liu F., Stehouwer J., Liu X., Jain A.K. On the detection of digital face manipulation. Proceedings of the IEEE/CVF Conference on Computer Vision and Pattern Recognition.

[32] Li X., Yu K., Ji S., Wang Y., Wu C., Xue H. Fighting against deepfake: Patch & pair convolutional neural networks (ppcnn). Proceedings of the Companion Proceedings of the Web Conference 2020.

[33] Charitidis P., Kordopatis-Zilos G., Papadopoulos S., Kompatsiaris I. (2020). A face preprocessing approach for improved deepfake detection. arXiv.

[34] Kumar A., Bhavsar A., Verma R. Detecting deepfakes with metric learning. Proceedings of the 2020 8th International Workshop on Biometrics and Forensics (IWBF).

[35] Khalil S.S., Youssef S.M., Saleh S.N. (2021). iCaps-Dfake: An integrated capsule-based model for deepfake image and video detection. Future Internet.

[36] Wodajo D., Atnafu S. (2021). Deepfake video detection using convolutional vision transformer. arXiv.

[37] Li Y., Chang M.C., Lyu S. In ictu oculi: Exposing ai created fake videos by detecting eye blinking. Proceedings of the 2018 IEEE International Workshop on Information Forensics and Security (WIFS).

[38] Sabir E., Cheng J., Jaiswal A., AbdAlmageed W., Masi I., Natarajan P. (2019). Recurrent convolutional strategies for face manipulation detection in videos. Interfaces.

[39] Wubet W.M. (2020). The deepfake challenges and deepfake video detection. Int. J. Innov. Technol. Explor. Eng..

[40] Singh A., Saimbhi A.S., Singh N., Mittal M. (2020). DeepFake video detection: A time-distributed approach. SN Comput. Sci..

[41] De Lima O., Franklin S., Basu S., Karwoski B., George A. (2020). Deepfake detection using spatiotemporal convolutional networks. arXiv.

[42] Masi I., Killekar A., Mascarenhas R.M., Gurudatt S.P., AbdAlmageed W. (2020). Two-branch recurrent network for isolating deepfakes in videos. Proceedings of the European Conference on Computer Vision.

[43] Montserrat D.M., Hao H., Yarlagadda S.K., Baireddy S., Shao R., Horváth J., Bartusiak E., Yang J., Guera D., Zhu F. Deepfakes detection with automatic face weighting. Proceedings of the IEEE/CVF Conference on Computer Vision and Pattern Recognition Workshops.

[44] Mehra A. (2020). Deepfake Detection Using Capsule Networks with Long Short-Term Memory Networks. Master’s Thesis.

[45] Nguyen X.H., Tran T.S., Nguyen K.D., Truong D.T. (2021). Learning spatio-temporal features to detect manipulated facial videos created by the deepfake techniques. Forensic Sci. Int. Digit. Investig..

[46] Redmon J., Divvala S., Girshick R., Farhadi A. You only look once: Unified, real-time object detection. Proceedings of the IEEE Conference on Computer Vision and Pattern Recognition.

[47] Chen W., Huang H., Peng S., Zhou C., Zhang C. (2020). YOLO-face: A Real-Time Face Detector. Vis. Comput..

[48] Szegedy C., Ioffe S., Banjouke V., Alemi A. Inception-v4, inception-resnet and the impact of residual connections on learning. Proceedings of the AAAI Conference on Artificial Intelligence.

[49] Srivastava N., Hinton G., Krizhevsky A., Sutskever I., Salakhutdinov R. (2014). Dropout: A simple way to prevent neural networks from overfitting. J. Mach. Learn. Res..

[50] Chen T., Guestrin C. Xgboost: A scalable tree boosting system. Proceedings of the 22nd ACM SIGKDD International Conference on Knowledge Discovery and Data Mining.

[51] Zhang L., Zhan C. Machine learning in rock facies classification: An application of XGBoost. Proceedings of the International Geophysical Conference.

[52] Hanley J.A., McNeil B.J. (1982). The meaning and use of the area under a receiver operating characteristic (ROC) curve. Radiology.

[53] Fawcett T. (2006). An introduction to ROC analysis. Pattern Recognit. Lett..

[54] He K., Zhang X., Ren S., Sun J. Deep residual learning for image recognition. Proceedings of the IEEE Conference on Computer Vision and Pattern Recognition.

[55] Chollet F. Xception: Deep learning with depthwise separable convolutions. Proceedings of the IEEE Conference on Computer Vision and Pattern Recognition.

[56] Tan M., Le Q. Efficientnet: Rethinking model scaling for convolutional neural networks. Proceedings of the International Conference on Machine Learning.

[57] Dozat T. Incorporating Nesterov Momentum into Adam; 2016. https://openreview.net/forum?id=OM0jvwB8jIp57ZJjtNEZ.

[58] Bonettini N., Cannas E.D., Mandelli S., Bondi L., Bestagini P., Tubaro S. Video face manipulation detection through ensemble of CNNs. Proceedings of the 2020 25th International Conference on Pattern Recognition (ICPR).

[59] Fradkin D., Muchnik I. (2006). Support Vector Machines for Classification.

[60] Staelin C. (2003). Parameter Selection for Support Vector Machines; HPL-2002-354R1.

[61] Xing Y., Lv C., Cao D. (2020). Advanced Driver Intention Inference: Theory and Design.

[62] Kaati L., Omer E., Prucha N., Shrestha A. Detecting multipliers of jihadism on twitter. Proceedings of the 2015 IEEE International Conference on Data Mining Workshop (ICDMW).

